# Highly Sensitive, Highly Specific Whole-Cell Bioreporters for the Detection of Chromate in Environmental Samples

**DOI:** 10.1371/journal.pone.0054005

**Published:** 2013-01-11

**Authors:** Rita Branco, Armando Cristóvão, Paula V. Morais

**Affiliations:** 1 IMAR, 3004-517 Coimbra, Portugal; 2 Escola Universitária Vasco da Gama, Mosteiro de S. Jorge de Milréu, Estrada da Conraria, Castelo Viegas – Coimbra, Portugal; 3 Center for Neuroscience and Cell Biology, University of Coimbra, Coimbra, Portugal; 4 Department of Life Sciences, FCTUC, University of Coimbra, Coimbra, Portugal; Missouri University of Science and Technology, United States of America

## Abstract

Microbial bioreporters offer excellent potentialities for the detection of the bioavailable portion of pollutants in contaminated environments, which currently cannot be easily measured. This paper describes the construction and evaluation of two microbial bioreporters designed to detect the bioavailable chromate in contaminated water samples. The developed bioreporters are based on the expression of *gfp* under the control of the *chr* promoter and the *chrB* regulator gene of Tn*OtChr* determinant from *Ochrobactrum tritici* 5bvl1. pCHRGFP1 *Escherichia coli* reporter proved to be specific and sensitive, with minimum detectable concentration of 100 nM chromate and did not react with other heavy metals or chemical compounds analysed. In order to have a bioreporter able to be used under different environmental toxics, *O. tritici* type strain was also engineered to fluoresce in the presence of micromolar levels of chromate and showed to be as specific as the first reporter. Their applicability on environmental samples (spiked Portuguese river water) was also demonstrated using either freshly grown or cryo-preserved cells, a treatment which constitutes an operational advantage. These reporter strains can provide on-demand usability in the field and in a near future may become a powerful tool in identification of chromate-contaminated sites.

## Introduction

Contamination of soils and water supplies with chromium compounds is considered a serious environmental issue. The extensive chromate utilization in industries has resulted in a large number of contaminated sites worldwide [1]. Nowadays, water-soluble heavy metal components, including chromate compounds, are of particular concern for drinking water and agricultural water quality [2]. Although many of these chromate compounds consist of complexes of low bioavailability and toxicity, they often persist in the environment and may be converted to more dangerous and bioavailable forms: the Cr(VI) free oxyanions.

Detection of the presence of low concentrations of chromate is indispensable to identify contaminated locations and to control the progress of remediation efforts. Analytic methods such as inductively coupled plasma atomic electron spectrometry (ICP/AES) or mass spectrometry (ICP/MS), flow injection atomic absorption (FIAAS) or electrochemical methods are the most used methods for quantification of total metals. These methodologies require the sample digestion with strong acids, which are pollutant, and are not able to distinguish between the bio-available and non-bio-available fractions of the metals. Therefore, the environmental risk for each metal is difficult to predict using these methodologies. [3], [4], [5]. On the other hand, the use of bacterial biosensors/bioreporters allows sensitive determinations of the bioavailable contaminant, without expensive equipment or specialized training. It provides a simple way of determining contaminants and some stress-induced biosensors report the mutagenic effects of samples with great sensitivity [6]–[8].

Microbial biosensors are increasingly being used as specific and sensitive sensing devices for measuring biologically relevant concentrations of pollutants [6], [7], [9]. Due to their low cost, lifespan, and range of fitting growth conditions (e.g. pH and temperature), microorganisms have been widely employed in the construction of biosensors.

In biosensor technology, in order to detect bioavailable environmental pollutants, the devices are constructed by fusing a pollutant-responsive promoter to a reporter gene. The reporter gene expression may be quantified and is a measure of the availability of a specific pollutant in the sample. Several field applications for these biosensors have been previously reported, such as quantification of polychlorinated biphenyls, alkanes, aromatic compounds, including polycyclic aromatic hydrocarbons (PAHs), biocides, antibiotics and heavy metals (reviewed in [10]–[12]).

In the case of heavy-metal detection, several bioreporters have been constructed to be used in general toxicity testing or in detection of specific metals, e.g. mercury, copper, lead, cadmium, nickel, zinc and arsenic [4], [13]–[17]. Despite the different metal-specific bacterial reporters available, only a few have been drawn for the analysis of environmental samples and even fewer used as chromium (Cr(VI) and Cr(III))-specific bacterial sensors in direct testing of soils [5]. A recombinant luminescent bacterial sensor for chromate was constructed based on a chromate resistance determinant located in megaplasmid pMOL28 of multiresistant *Ralstonia metallidurans* CH34 [18]. However, this bacterial reporter revealed to have its response affected by Cr(III) ions, and therefore, to be a system not completely selective to chromate.

Commonly used reporter systems include *lacZ* for β-galactosidase activity, *luxAB* and *lucFF* for bioluminescence measurements [19], [20]. The gene for green fluorescent protein (GFP) is increasingly used to construct biosensors, because it allows for in situ assessment of elemental bioavailability and can report relevant concentrations of pollutants [21]–[23].

In the present study we describe the construction and characterization of a chromate-GFP bioreporter *E. coli*. This bioreporter was constructed by creating a transcriptional fusion between *gfp* gene and the genetic unit composed by the promoter and the chromate regulatory gene (*chrB*) from Tn*OtChr* of *Ochrobactrum tritici* 5bvl1. The characterized Tn*OtChr* element, carrying *chr* operon of *O. tritici* 5bvl1, contains a regulatory gene (*chrB*) and three additional chromate resistance genes (*chrA*, *chrC* and *chrF*) [24], [25]. In order to have a bioreporter resistant to other toxics and able to detect chromate in multi-contaminated environmental samples, we also engineered the type strain of *O. tritici* to become fluorescent in the presence of toxic levels of chromate. This strain was chosen since it is an environmental strain [26] and its bacterial genus, *Ochrobactrum,* is widely distributed, being able to survive in different environments, including habitats where contamination with several compounds is present [27]–[29]. This great diversity, including habitats contaminated with chromate, suggests that environmental whole-cell bioreporter could be a great solution for chromate detection across a wide range of conditions.

## Materials and Methods

### Ethics Statement

No specific permits were required for the described field studies. The water samples were collected in a public river beach open to the public (Mondego) and in 3 other public stream waters (Malhou, Braças and Carritos). Only waters were used for analysis in the present study.

### pCHRGFP1 *E. coli* Reporter Construction

**Figure 1:**
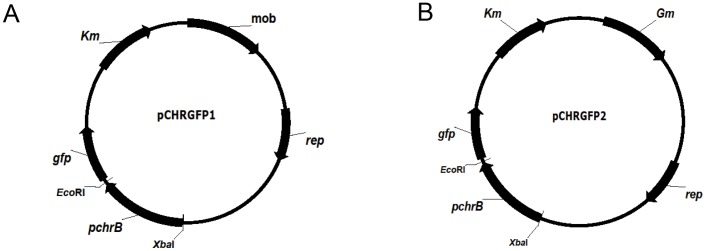
Schematic diagram of the plasmids engineered for this work. (A) Plasmid pCHRGFP1: pPROBE-NT containing the *chr* promoter and *chrB* gene upstream of *gfp*. (B) Plasmid pCHRGFP2: pCHRGFP1 with an additional gentamicin resistance gene cloned into *mob* gene. Arrows indicate the direction of transcription. The restriction enzymes indicated were those used in this work.

The reporter plasmid was constructed by inserting a heavy metal responsive element from a natural resistance mechanism into a plasmid-containing promoterless green fluorescence protein (*gfp*) gene. Standard recombinant techniques were used for the construction of this chromate sensor plasmid [30]. The genomic region containing the *chr* promoter and *chrB* gene was amplified from strain *O. tritici* 5bvl1 DNA with primers Pchr1 (5′-CG***TCTAGA***GATTGCTTATTCCTATTGCCA-3′) and Pchr2 (5′-CG***GAATTC***TCATACGCTGAGGGTCCCTTT-3′), containing engineered *Xba*I and *Eco*RI recognition sites (restriction sites are italicized and in bold), following the standard PCR conditions. The resulting PCR product (1.11 Kbp) was gel purified prior to being cloned into pGEM-T plasmid (Promega). DNA sequencing was performed to verify that the correct amplification product was obtained prior to digestion by *Xba*I and *Eco*RI and transference of the restriction digestion product into the multiple cloning site of pPROBE-NT [31]. This construct was then transformed into *Escherichia coli* DH5α resulting in a pCHRGFP1 *E. coli* chromate reporter (Figure 1A).

### pCHRGFP2 *O. tritici* Reporter Construction

The previous reporter plasmid was engineered by introducing an additional antibiotic resistance cassette. After gentamicin resistance gene amplification from pBBR1MCS-5 plasmid [32], the PCR fragment was cloned downstream into *Not*I site of previous construct (Figure 1B). This plasmid was then inserted into *O. tritici* type strain by electroporation. Selection of plasmid-expressing clones was performed on LB plates with gentamicin (15 µg/ml). One clone was considered a pCHRGFP2 *O. tritici* reporter and was subjected to further analyses.

### Reporter Activity Assays

The reporter strains pCHRGFP1 *E. coli* and pCHRGFP2 *O. tritici* were grown overnight, in a shaker (200 rpm) at 30°C in Luria–Bertani (LB) medium and used as inoculums. Cultures of the bioreporters used in the experiments (except when otherwise stated) were performed in TMM medium (Tris-buffered mineral salts medium [33] supplemented with glucose 0.5% and vitamins [24]), incubated at 37°C, and grown to an OD600nm of about 0.2–0.3, corresponding to cells on exponential phase of growth. Frozen cells at −80°C were prepared by resuspending exponential growing cells in TMM medium with 15% glycerol. To study the specificity and sensitivity of the bioreporters, these exponential cells were stressed by addition of the indicated concentration of chromate (as K_2_CrO_4_), chromium (III) chloride, arsenate, phosphate, sulphate, molybdate, tungstate, nickel, cobalt, cadmium, copper, and zinc. The stock metal solutions (1 M) were prepared by dissolving analytical grade (>99.0%) metal salts in ultra pure water sterilized by filtration. The stressed cultures were then incubated at 37°C, for the period of 5 hours. During this period, reaction volumes of 200 µl were transferred onto white 96-well plates and GFP fluorescence intensity was measured hourly and in triplicate.

The effect of the growth media composition in the ability of the pCHRGFP1 *E. coli* reporter to measure chromate was also tested using two different media. Exponential cells grown in the chemically defined medium TMM, and in the rich medium LB, were subjected to increasing chromate concentrations. To assess the effect of culture growth phase on bioreporter response, cells grown overnight, corresponding to cells at stationary phase, in LB or in TMM, were exposed to different concentrations of chromate and the results compared to the ones obtained by using cells in exponential growth phase.

Environmental analyses with Mondego river water were performed by mixing (1∶1) exponential growing or frozen cells of pCHRGFP1 *E. coli* and pCHRGFP2 *O. tritici* reporters with natural river water (uncontaminated sample) or river water spiked with 1 µM and 10 µM of chromate (contaminated sample). Assays with environmental waters from Malhou, Braças and Carritos were performed as described above using only exponential growing cells.

Optical density and fluorescence were measured, in replicate experiments, during the incubation periods.

### Spectrofluorometry

Fluorescence intensity was measured with a Gemini EM Fluorescence Microplate Reader (Molecular Devices), with emission, excitation and cutoff wavelengths at 510, 480 and 595 nm, respectively. Relative fluorescence unit (RFU) is defined as the culture fluorescence relative to culture biomass at OD600nm SpectraMax Plus384 Absorbance Microplate Reader (Molecular Devices). Induction ratio is defined as the RFU of an effector-exposed sample divided by the RFU of a no-effector control (0 µM).

### Epifluorescent Microscopy

Bioreporter cells were visualized under a Leitz Laborlux K epifluorescent microscope (Leica) equipped with a 50 W mercury lamp, a BP 450–490 excitation filter and a LP 515 nm emission filter. Overnight chromate stressed cells were placed onto a microscope slide and examined under the microscope for GFP fluorescence expression.

## Results

### Genetic Description of the Bacterial Bioreporters

The *chr* promoter and the *chrB* gene of the *chr* resistance determinant of strain *O. tritici* 5bvl1 (Tn*OtChr*) was cloned into the broad-host-range vector pPROBE-NT [31], upstream from the *gfp* gene, creating a p*chrB*_*gfp* transcriptional fusion. One bioreporter was created when *E. coli* DH5α cells were transformed with this construct, which resulted in the bioreporter designated by pCHRGFP1 *E. coli* (Fig. 1A). An additional gentamicin gene was inserted into *mob* gene of pCHRGFP1 resulting in pCHRGFP2 (Fig. 1B), which was then introduced into *O. tritici* type strain. This second bioreporter was named by pCHRGFP2 *O. tritici*.

### Detection of Chromate by Bioreporter Strains

**Figure 2:**
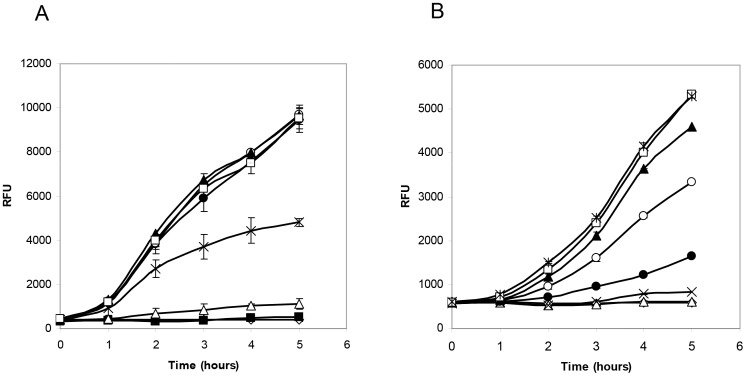
Time-dependent induction of pCHRGFP1 *E. coli* (A) and pCHRGFP2 *O. tritici* (B) reporters with chromate. Cells were exposed to increasing concentrations of chromate, 0 nM (⋄), 10 nM (▪), 100 nM (▵), 1 µM (x), 5 µM (•) 10 µM (○), 25 µM (▴), 50 µM (□) e 100 µM (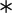). The data are the mean values of three experiments with the standard deviations.

**Figure 3:**
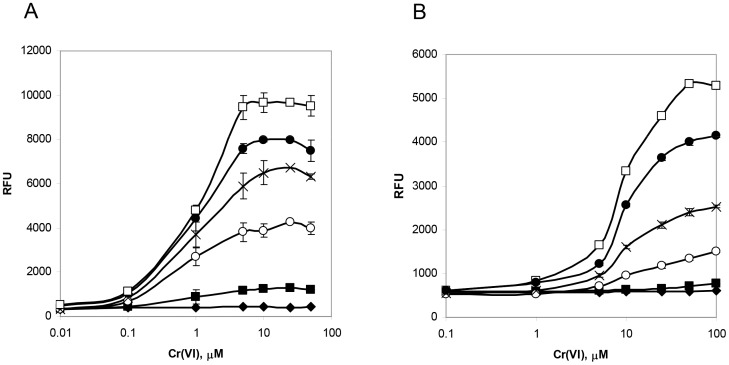
Chromate-fluorescence response of pCHRGFP1 *E. coli* (A) and pCHRGFP2 *O. tritici* (B) reporters measured after different exposure periods. 0 h chromate incubation (⧫), 1 h chromate incubation (▪), 2 h chromate incubation (○), 3 h chromate incubation (x), 4 h chromate incubation (•), 5 h chromate incubation (□). The data are the mean values of three experiments with the standard deviations.

Cultures of pCHRGFP1 *E. coli* and pCHRGFP2 *O. tritici* were grown under a range of chromate concentrations (0.01 to 100 µM Cr(VI)), to assess whether functional GFP was produced and to measure the bioreporter sensitivity. The fluorescence data recorded from exponential growth cells of both strains in TMM, with and without chromate, under aerobic conditions are shown in Fig. 2 and Fig. 3. The results indicated that cells were able to produce functional GFP when subjected to chromate.

Time-dependent induction of the two bacterial reporters in response to chromate was determined by incubating periodically the cells with the metal ion. The induction of green fluorescence production by both bioreporters when exposed to chromate showed a time-dependence (Fig. 2). As shown in this figure, as the time of induction increased, there was an increase in the fluorescence emitted by bacterial strains. The profile of both bioreporters response showed that during the period of cell incubation, the specific fluorescence intensity continuously increased. Although the maximum signal for these reporters was reached after 5 h of exposure to chromate, the incubation time could be certainly reduced to 3 h since the fluorescence signal obtained during this period of time was high enough to reach suitable conclusions, as it is possible to observe in Fig. 3.

The intensity of the GFP fluorescence emission of pCHRGFP1 *E. coli* and pCHRGFP2 *O. tritici* was found to be dependent on the concentration of chromate ions in the growth medium, given that the increase of chromate concentrations under the same incubation conditions resulted in increasing fluorescence (Fig. 2 and Fig. 3). The lowest concentration of chromate that caused a noticeable response by pCHRGFP1 reporter was 0.1 µM Cr(VI), with a fluorescence intensity four times higher than the control experiment after 5 h of incubation (Fig. 2A). The intensity of signal fluorescence increased along with the amount of chromate concentration, up to 10 µM (Fig. 3A), after which the fluorescence reached a plateau. When chromate concentration exceeded 50 µM, green fluorescence started to decrease slightly. This was possibly due to the toxic effect of chromium ions on the bacterial cells. The second bioreporter, pCHRGFP2 *O. tritici*, was not as sensitive to chromate as pCHRGFP1. Higher chromate concentrations (at least 1 µM) were needed to induce a clear green fluorescence production by pCHRGFP2 (Fig. 2B and Fig. 3B). These reporter assays also showed maximum fluorescence intensity values lower than those achieved by using the pCHRGFP1 *E. coli* bioreporter. Moreover, the saturation of fluorescence signal was achieved for higher chromate concentrations (>50 µM).

No GFP fluorescence emission could be detected in the parent strains *E. coli* DH5α and *O. tritici* carrying the empty pPROBE-NT plasmid (data not shown). In addition, Fig. 2 and Fig. 3 show that the background fluorescence exhibited by the untreated bioreporters (cells without chromate) did not change or oscillate during the incubation period.

### Selectivity of the Chromate Bioreporters

The selectivity of the pCHRGFP1 *E. coli* and pCHRGFP2 *O. tritici* bioreporters to metal ions was also evaluated. The reporter cells were treated with increasing concentrations of the different divalent metal ions. The levels of fluorescence measured during incubation (5 h) are shown in Table 1. The metals Cd(II), Zn(II), Co(II), Ni(II) and Cu(II) did not induce significant changes (less than 1%) in green fluorescence compared to control.

**Table 1:** Induction of fluorescence in both bioreporters by several compounds.

Compound	Bioreporter	Concentration(µM)	Indutionratio
	pCHRGFP1 *E. coli*	10	0.9±0.03
		100	1.2±0.12
Ni^2+^	pCHRGFP2 *O. tritici*	10	1.2±0.08
		100	1.1±0.02
	pCHRGFP1 *E. coli*	10	0.7±0.02
		100	0.7±0.1
Co^2+^	pCHRGFP2 *O. tritici*	10	0.8±0.02
		100	0.7±0.04
	pCHRGFP1*E. coli*	10	0.8±0.01
		100	0.9±0.15
Cd^2+^	pCHRGFP2 *O. tritici*	10	0.9±0.03
		100	0.8±0.07
	pCHRGFP1 *E. coli*	10	0.7±0.03
		100	0.8±0.03
Zn^2+^	pCHRGFP2 *O. tritici*	10	0.7±0.05
		100	0.8±0.03
	pCHRGFP1 *E. coli*	10	0.9±0.03
		100	1.1±0.02
Cu^2+^	pCHRGFP2 *O. tritici*	10	0.9±0.08
		100	1.0±0.02
	pCHRGFP1 *E. coli*	100	0.9±0.02
		1000	0.9±0.04
MoO_4_^2^	pCHRGFP2 *O. tritici*	100	1.0±0.03
		1000	0.9±0.05
	pCHRGFP1 *E. coli*	100	1.0±0.01
		1000	1.0±0.04
WO_4_^2-^	pCHRGFP2 *O. tritici*	100	1.1±0.01
		1000	0.9±0.08
	pCHRGFP1 *E. coli*	10	1.1±0.04
		100	1.4±0.01
		1000	1.3±0.01
Cr (III)	pCHRGFP2 *O. tritici*	10	1.0±0.01
		100	1.1±0.02
		1000	1.0±0.02

**Figure 4:**
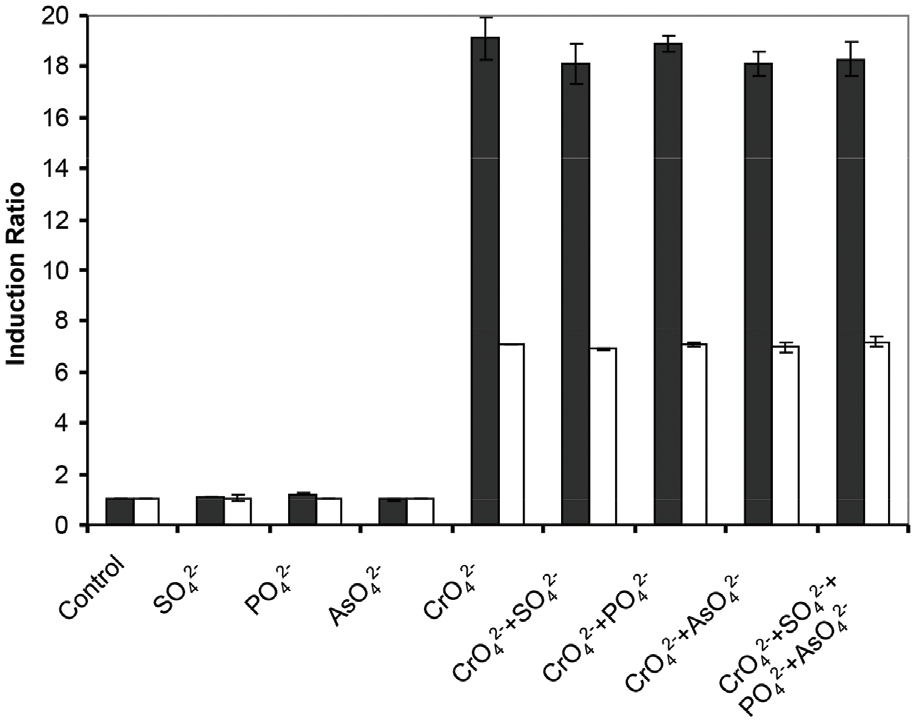
Selectivity of the pCHRGFP1 *E. coli* and pCHRGFP2 *O. tritici* reporters to different compounds. Cells were treated individually with sulphate and phosphate in concentration of 1 mM and with arsenate and chromate in concentration of 0.1 mM or mixtures of the several compounds for 5 hours. Control refers to no-metal treatment reporter bacteria. The data are the mean values of three experiments with the standard deviations.

Since structural configuration of chromate is comparable to arsenate, phosphate sulphate, molybdate and tungstate, both bioreporter cells were also treated with these compounds in order to evaluate their specificity to chromate. The concentrations of the compounds tested ranged from 10 µM to 10 mM, in order to ensure that, if the compound was an inducer, an induction concentration was tested. In the case of arsenate, lower concentrations (1 to 100 µM) were used because higher concentrations have demonstrated to be significantly inhibitory to cell growth. As it was observed in the tests using metals different from chromate, pCHRGFP1 *E. coli* or pCHRGFP2 *O. tritici* did not respond to any of the effectors, at any concentration tested (Table 1 and Fig. 4).

Although pCHRGFP1 *E. coli* and pCHRGFP2 *O. tritici* bioreporters did not respond to the several effectors analysed, the contaminated sites often contain mixtures of metal contaminants and other compounds. Thus, the effect of the presence of multiple effectors on reporters response was also assessed. The combinations of sulphate, phosphate or arsenate with chromate did not change the induction ratio observed in the individual test (with chromate only) (Fig. 4). Based on these induction results and despite of the lower chromate sensitivity of *O. tritici* reporter when compared with *E. coli* reporter, both bioreporter cells behaved as efficient chromate selective reporter systems.

### Growth Phase and Medium Dependence on Bioreporter Cells

**Figure 5:**
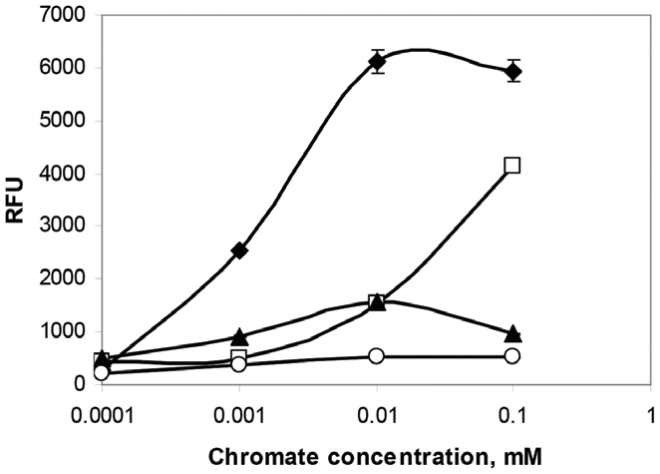
Effect of growth phase and culture medium on efficiency of pCHRGFP1 *E. coli* reporter. Exponential cells grown in TMM medium (⧫) or LB medium (□) and stationary cells grown in TMM medium (▴) or LB medium (○) were incubated with the indicated chromate concentrations for 5 hours. The data are the mean values of three experiments with the standard deviations.

It is known that growth-phase and growth-medium can affect a reporter gene expression in a whole-cell biosensor. To assess this problem, the expression of *gfp* by pCHRGFP1 *E. coli* was studied under different bacterial growth conditions. In the experiments with exponential growth chromate-treated cells, the kinetics of GFP formation essentially matched those of biomass formation, with the net fluorescence of the culture increasing during the exponential phase of growth. Chromate induced *gfp* expression in cells grown in both media examined. However, the level of fluorescence was different between both growth media used: although the bioreporter did respond to chromate when cultivated in the rich medium, the induction ratio was only 25% of that obtained in TMM medium (Fig. 5). Cells obtained from overnight LB or TMM growth cultures (stationary phase) exhibited a fluorescence level much lower than the fluorescence signal obtained with exponential growth cells. These results confirmed that growth phase affects induction of the chromate bioreporter.

### Chromate Bioreporters in Environmental Samples

**Figure 6:**
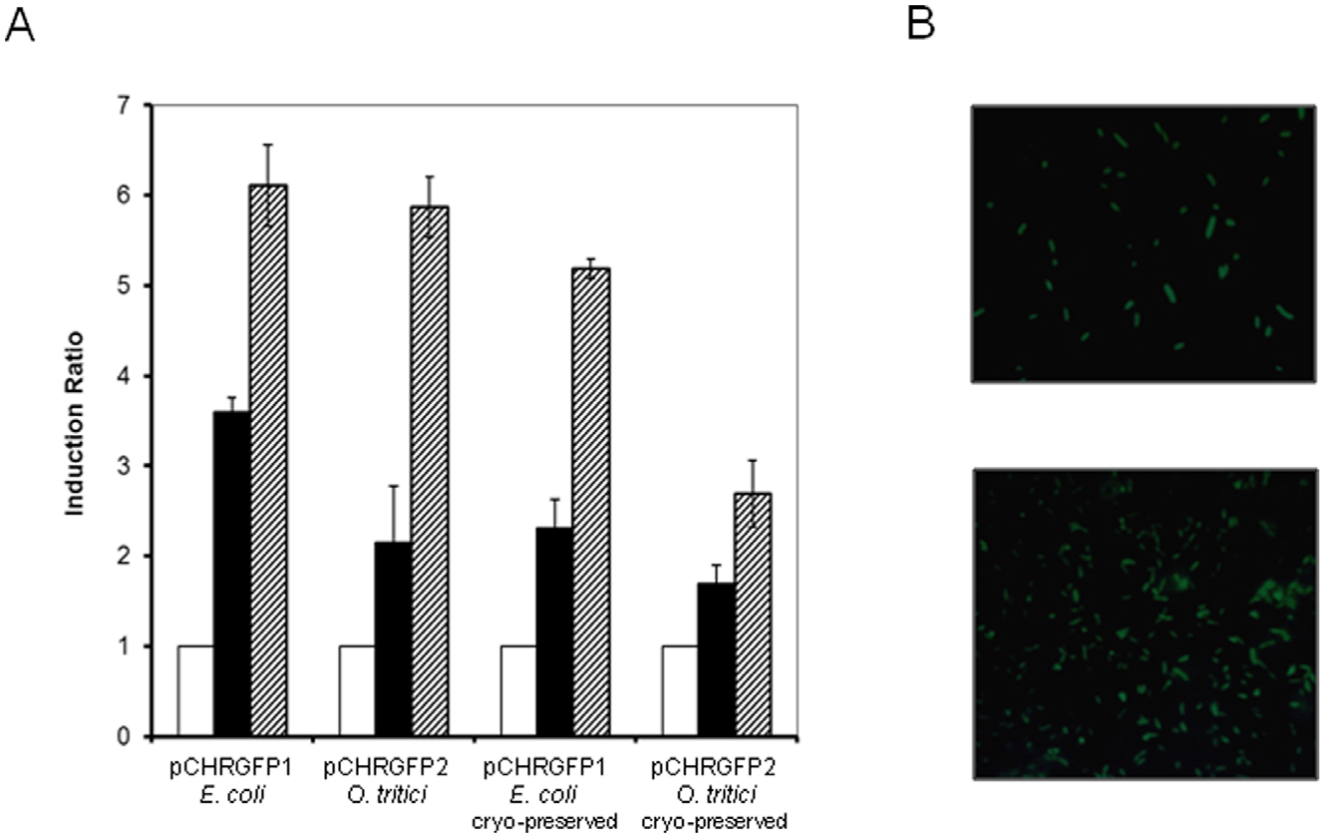
GFP reporter detection of chromate-contaminated environmental water samples. **A)** Cultures of fresh exponential-growing cells or cryo-preserved exponential cells of pCHRGFP1 *E. coli* and pCHRGFP2 *O. tritici* reporters were exposed to Mondego river water (white bars), Mondego river water spiked with 1 µM chromate (black bars) and 10 µM chromate (dashed bars). The fluorescence was assayed after 5 hours of incubation. The data are the mean values of three experiments with the standard deviations. **B)** Exponential grown cells of pCHRGFP1 *E. coli* (upper panel) and of pCHRGFP2 *O. tritici* (lower panel) were incubated overnight to Mondego river water spiked with 10 µM of chromate, after which green fluorescent protein expression in bacterial cells was visualized by using epifluorescence microscope. Magnification, ×1000.

**Figure 7:**
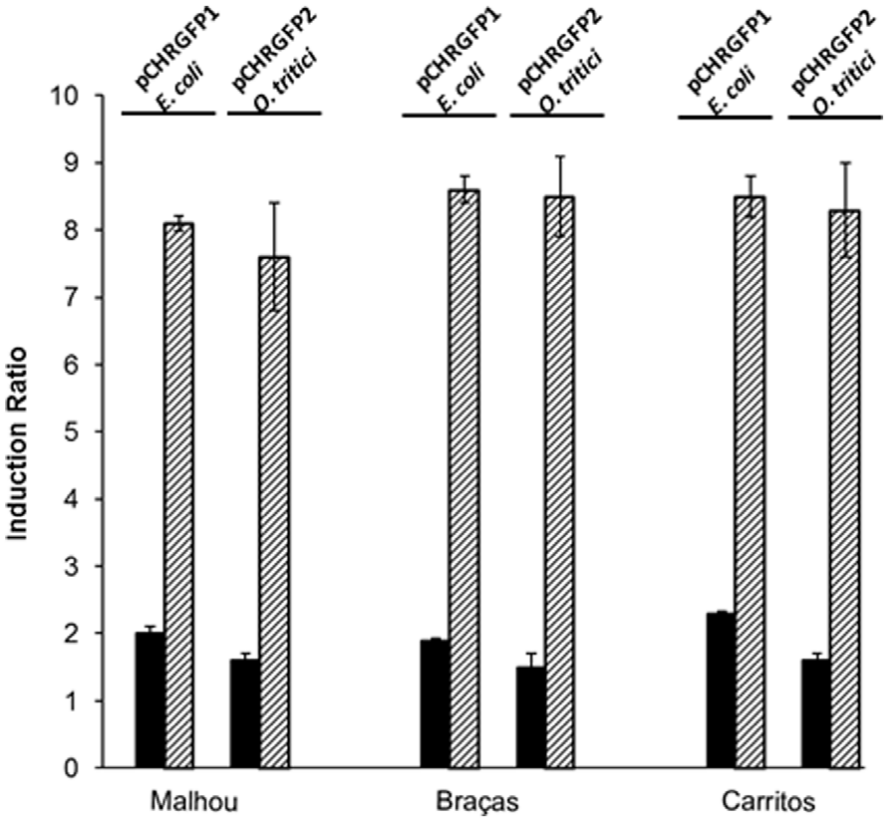
Fluorescence of bioreporter strains exposed to different chromate-contaminated environmental waters. Cultures of exponential-growing cells of pCHRGFP1 *E. coli* and pCHRGFP2 *O. tritici* were exposed to chromate spiked stream waters of Malhou, Braças and Carritos. These samples were contaminated with 1 µM chromate (black bars) and 10µM chromate (dashed bars). The fluorescence was assayed after 5 hours of incubation. The data are the mean values of three experiments with the standard deviations.

Both bioreporter strains, pCHRGFP1 *E. coli* and pCHRGFP2 *O. tritici,* were used to determine chromate concentration in environmental water samples with differentchemical composition (Table 2). Waters differed mainly in their content of iron, chlorides, magnesium, selenium and barium. Portuguese river water samples, contaminated with 1 µM and 10 µM of chromate, induced GFP expression in exposed fresh cells and the green fluorescence intensity was time and dose dependent (Fig. 6 and Fig. 7). Additionally, cryo-preserved reporter cells were tested since they might be considered a useful for operational reasons, allowing their storage and use over time. In order to assess their activity, both bioreporter frozen cells were exposed to chromate contaminated environmental Mondego river water samples and showed to be able to produce GFP, when submitted to contaminated waters. Figure 6A shows that fresh exponential growing cells were more efficient than the frozen cells since the fluorescence levels were higher than those obtained by cryo-preserved cells. This data clearly demonstrates that strains distinguished chromate contaminated water of uncontaminated water even when used after freezing.

**Table 2:** Chemical analyses of environmental water samples.

Compound (mg/l)	Water Samples			
	Mondego	Malhou	Braças	Carritos
Nitrate	*<11	<11	<11	<11
Chloride	10	47	36.5	70.4
Phosphate	<0.14	<0.14	<0.14	<0.14
Sulfate	<15	<15	<15	<15
Phenol	<0.001	n.d.	<0.001	<0.001
Hydrocarbon	<0.01	<0.01	<0.01	<0.01
Fluoride	<0.3	0.73	<0.3	<0.3
Cyanide	<0.01	n.d.	<0.01	<0.01
Iron	0.09	<0.04	4.0	0.1
Magnesium	0.02	4.9	0.06	0.01
Copper	<0.01	n.d.	<0.01	<0.01
Zinc	<0.1	<0.1	<0.1	<0.1
Arsenic	<0.003	<0.003	<0.003	<0.003
Boron	<0.2	0.08	<0.2	<0.2
Cadmium	<0.0015	<0.0015	<0.0015	<0.0015
Chromium	<0.006	0.016	<0.006	<0.006
Lead	<0.006	<0.006	<0.006	<0.006
Selenium	0.001	0.0023	0.0026	0.001
Barium	0.02	n.d.	0.03	0.06
Mercury	<0.0004	<0.0004	<0.0004	<0.0004
**pH**	7.9	7.9	6.2	6.4
**Dissolved oxygen (%)**	97	80	41	84

* limit of detection.

n.d.: not determined.

The different composition of the environmental waters tested did not affect the fluorescence signals (Fig. 7). Additionally, the only stream water, Malhou, which naturally contained 0.3 µM of total chromium, showed an increase of 1.5 fold comparatively to the other waters for the non-contaminated assays.

When examined by fluorescence microscopy, the image of the bacterial cultures of pCHRGFP1 *E. coli* and pCHRGFP2 *O. tritici* incubated overnight with chromate contaminated waters (10 µM chromate), clearly shows induction of fluorescence, revealed by the presence of green cells (Fig. 6B). Incubation of both reporter cells with non-contaminated environmental water resulted in cells without fluorescence and therefore no green color could be detected on images of epi-fluorescence (data not shown).

## Discussion

In the present work, two whole-cell chromate reporters that can detect the presence of nanomolar amounts of chromate in laboratorial medium assays or micromolar concentrations of chromate in environmental samples were constructed. After the successful experiments with the pCHR*lacZ* fusion reporter [24], we concluded that ChrB could be used as a chromate-sensing system. To take advantage of this ability, a reporter construct was performed by placing a promoterless *gfp* gene under the control of a genetic fragment composed by a *chr* promoter and *chrB* gene of *O. tritici* 5bvl1.

Several bacterial bioreporters for different environmental pollutants have been developed using various reporter genes, such as *lacZ, luxAB,* and *lucFF* (reviewed in 13, 34). Although the colorimetric enzyme assay and bioluminescence have been very successful as reporters for detection of several compounds, such as metals, metalloids or aromatic compounds, these methods require the addition of exogenous substrates or cofactors. Moreover, these assays require additional experimental steps, such as centrifugation, cell lysis, pH adjustment, before enzyme activity measurements. Regarding biological chromate sensing, few efforts have been carried out to achieve an efficient chromate biosensor. The few cases reported are based on fusion of chromate resistance genes with *lux* reporter gene [5], [18]. However, this system showed interactions among induction of the *chr* resistance determinant, chromate reduction, chromate accumulation, and sulfate concentration of the growth medium.

The use of *gfp* as a reporter gene gives these sort of biosystems some advantages such as the ability to use a simple and clean method without the need for exogenous enzyme substrates or other chemical compounds. Additionally, the ability to use fluorometry and fluorescence microscopy to examine GFP expression allows assured results for assessing the effectors bioavailability [22], [35].

Under uninduced conditions, fluorescence from pCHRGFP1 *E. coli* or pCHRGFP2 *O. tritici* was almost null, most probably because of the transcriptional shielding of the *pchrB*-*gfp* fusion. The ability to detect induction after moderately short incubation periods (3 hours) and at low inducer concentrations, nanomolar scale for pCHRGFP1 *E. coli* reporter and 1 µM for pCHRGFP2 *O. tritici* reporter, may reflect the fact that the *pchrB*-*gfp* fusion is a stable and well-performed construct, carried on an efficient plasmid that allows a high amplification of the signal, and also reflects the sensitivity of fluorescence detection instrumentation.

Both whole-cell reporters demonstrated ability to discriminate environmental water samples contaminated with chromium from uncontaminated samples. The notable ability to detect chromate levels lower than US or EU limit value for chromate concentrations in drinking water, 0.1 mg/l [36] or 0.05 mg/l [37] respectively make this system a hopeful successful tool to monitor the levels of chromate in environmental samples. Currently, many bacterial reporters or sensors have been reported and are considered promising applications in the fields of biotechnology and environmental sciences [7]. The development of portable biosensors able to be applied on-site to monitor environmental pollution is an urgent demand. For example, arsenic contamination of groundwater is a serious problem in several countries and sensitive biosensors were already tested with arsenite contaminated waters from these countries [38], [39]. A major characteristic of the whole-cell chromate biosensing systems developed in this study are their insensitivity to micromolar or millimolar levels of phosphate, sulphate, arsenate, molybdate, tungstate and diverse divalent metals. This is a noteworthy point, since environmental samples are composed of mixtures of compounds. In contaminated sites, the presence of many different chemicals other than the inducer compounds or environmental parameters, such as extreme salinity and pH values, could be toxic to or interfere with whole-cells, thereby causing inhibitory effects. Although it is impossible to determine the effect of all hypothetical components that could be present in an environmental sample, the bacterial reporters based on pCHRGFP represent a rapid, easy to perform, and inexpensive alternative to the conventional chemical method for chromate detection and measurement. It should also be noted that the chromate environmental bioassays required only small volumes of bacteria and the use of frozen cells did not impair its effectiveness. In the future, the optimization of this system, which can include the reconstitution of reporter strains from lyophilized powder, could greatly improve its applicability in the field as it has already been referred for other bioreporters [21], [40]. These bacterial bioreporters or their improved versions could complement or even replace traditional analytical methods, providing with confidence data that could be useful in risk assessment and evaluation of the remediation needs of chromate contaminated sites.

### Conclusions

The most innovative aspect of this paper is the demonstration of the high specificity and high sensitivity of two new chromate bioreporters constructed by our team. This paper includes the construction, laboratory characterization, and environmental sample testing of pCHRGFP1 *E. coli* and pCHRGFP2 *O. tritici* reporters for the detection of chromate. In fact, the pCHRGFP1 *E. coli* reporter has a very low detection limit enabling it to be used to evaluate chromate in potable waters. The most important advantage of these bioreporters is the fact it is a sensing system with a highly specific chromate selectivity, thus making possible to measure chromate among several compounds. In consequence, since field samples often contain unknown components, these bioreporters could be useful in the screening of chromate among unidentified contaminants in the environment. Moreover, chromium can be in the environment in different oxidation states which are not all bioavailable. The GFP-based bacterial reporter systems can provide information about the bioavailability of this metal, which is the most relevant information in environmental risk assessment and the potential biological impact of a contaminant [34].
